# Let-7c down-regulation in *Helicobacter pylori*-related gastric carcinogenesis

**DOI:** 10.18632/oncotarget.6642

**Published:** 2015-12-17

**Authors:** Matteo Fassan, Deborah Saraggi, Laura Balsamo, Luciano Cascione, Carlo Castoro, Irene Coati, Marina De Bernard, Fabio Farinati, Vincenza Guzzardo, Nicola Valeri, Carlo Federico Zambon, Massimo Rugge

**Affiliations:** ^1^ Department of Medicine (DIMED), Surgical Pathology & Cytopathology Unit, University of Padua, Padua, Italy; ^2^ Institute of Oncology Research and Swiss Institute of Bioinformatics, Lymphoma & Genomics Group, Bellinzona, Switzerland; ^3^ Istituto Oncologico Veneto, IOV-IRCCS, Surgery Unit, Padua, Italy; ^4^ Department of Biology, University of Padua, Padua, Italy; ^5^ Department of Surgical Oncology and Gastroenterology (DiSCOG), Gastroenterology Unit, University of Padua, Padua, Italy; ^6^ Molecular Pathology Division, Institute of Cancer Research, London and Sutton, UK; ^7^ Department of Medicine (DIMED), Clinical Pathology Unit, University of Padua, Padua, Italy

**Keywords:** microRNA, gastric adenocarcinoma, Helicobacter pylori, preneoplastic lesions

## Abstract

Aberrant let-7c microRNA (miRNA) expression has been observed in *Helicobacter pylori*-related gastric cancer (GC) but fragmentary information is available on the let-7c dysregulation occurring with each phenotypic change involved in gastric carcinogenesis. Let-7c expression was assessed (qRT-PCR) in a series of 175 gastric biopsy samples representative of the whole spectrum of phenotypic changes involved in *H. pylori*-related gastric oncogenesis including: i) normal gastric mucosa, as obtained from dyspeptic controls (40 biopsy samples); ii) non-atrophic gastritis (40 samples); iii) atrophic-metaplastic gastritis (35 samples); iv) intra-epithelial neoplasia (30 samples); v) GC (30 samples). Let-7c expression was also tested in 20 biopsy samples obtained from 10 patients before and after *H. pylori* eradication therapy (median follow-up: 10 weeks; range: 7-14). The results obtained were further validated by *in situ* hybridization on multiple tissue specimens obtained from 5 surgically treated *H. pylori*-related GCs. The study also included 40 oxyntic biopsy samples obtained from serologically/histologically confirmed autoimmune gastritis (AIG: 20 corpus-restricted, non-atrophic; 20 corpus-restricted, atrophic-metaplastic). Let-7c expression dropped from non-atrophic gastritis to atrophic-metaplastic gastritis, intra-epithelial neoplasia, and invasive GC (*p*<0.001). It rose again significantly following *H. pylori* eradication (*p*=0.009). As in the *H. pylori* model, AIG also featured a significant let-7c down-regulation (*p*<0.001). The earliest phases of the two pathways to gastric oncogenesis (*H. pylori*-environmental and autoimmune host-related) are characterized by similar let-7c dysregulations. In *H. pylori* infection, let-7c down-regulation regresses after the bacterium's eradication, while it progresses significantly with the increasing severity of the histological lesions.

## INTRODUCTION

Gastric cancer (GC) is a leading cause of cancer-related death worldwide [1, 2], and about 90% of non-cardia GCs are the ultimate consequence of longstanding *Helicobacter pylori* (*H. pylori*) infection [3–7]. *H. pylori* is the main trigger of Correa's multistep gastric carcinogenic cascade [8]. The stepwise changes occurring in the gastric mucosa have been well characterized, starting from longstanding inflammation, which may result in atrophic changes. Gastric mucosal atrophy is the “cancerization field” in which intra-epithelial neoplasia (IEN), and invasive cancer can develop [3, 9, 10].

In a minority of cases, the initiating cause of the mucosal inflammation is not environmental (*H. pylori*), but host-related (autoimmune gastritis [AIG]) [11, 12]. Autoimmune, corpus-restricted gastritis may result in mucosal atrophy too, and a higher risk of GC has been associated with AIG as well. The epidemiological and clinico-biological profiles of this “alternative” oncogenic pathway are less well defined than those of the *H. pylori-related* model [9, 13].

Despite recent extensive investigations on the molecular landscape of GC [14], the molecular grounds for the various steps in *H. pylori-*related carcinogenesis have yet to be fully elucidated, and no consistent and reliable biomarkers have become available for use in GC secondary prevention strategies [9, 15, 16].

Aberrant microRNA (miRNA) expression has consistently been reported in both *H. pylori* infection and *H. pylori-*related GC [17–19], but the miRNA dysregulation(s) associated with each of the phenotypic changes sequentially occurring in *H. pylori-*related carcinogenesis remain elusive. Among other miRNAs, the let-7 family members have reliably been found down-regulated in association with both *H. pylori-*associated gastritis [20, 21], and gastric cancer [22, 23]. Moreover, a significant down-regulation of let-7c (a let-7 family member) consistently occurs in the natural history of Barrett-adenocarcinoma (which features remarking similarities with *H. pylori-*related gastric carcinogenesis) [24].

This study focused on the involvement of let-7c over the whole series of non-neoplastic and neoplastic phenotypic changes occurring in *H. pylori-*infected gastric mucosa. Let-7c expression was profiled by qRT-PCR in a large series of formalin-fixed paraffin-embedded (FFPE) biopsy samples. The results obtained were then confirmed by miRNA *in situ* hybridization on surgical tissue specimens, and validated by exploring the publicly-available NCBI-GEO and TCGA databases.

## RESULTS

### Let-7c is down-regulated in *Helicobacter pylori*-related gastritis

**Figure 1:**
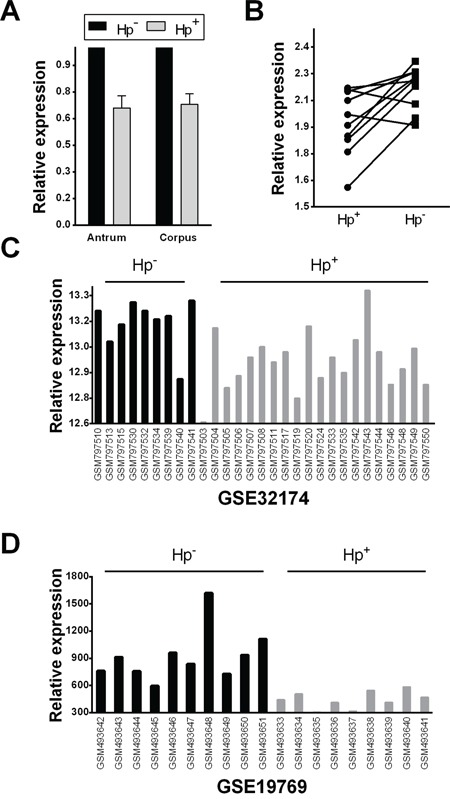
Let-7c is significantly down-regulated in *Helicobacter pylori* infection **A.** Let-7c expression was analyzed by qRT-PCR in a series of 40 samples of normal gastric mucosa and 40 of *H. pylori-related* gastritis. The latter showed a significantly down-regulated let-7c expression in both gastric compartments (antrum and corpus; *p*<0.001; relative expression= fold changes compared to normal mucosa samples set as 1.0). **B.** Let-7c expression levels were significantly up-regulated after *H. pylori* eradication in 8/10 patients (*p*=0.009; relative expression= distribution of normalized data in the two classes). **C–D.** Two independent studies in the NCBI GEO database showed a significant down-regulation of let-7c in *H. pylori-positive* samples (GSE32174 [31], adjusted *p*=3.47E-02; and GSE19769 [20], adjusted *p*=4.3E-04; relative expression= distribution of normalized data obtained from the GEO database).

In 80 biopsy samples (from 40 *H. pylori*-positive and 40 *H. pylori*-negative subjects), a significant let-7c down-regulation (qRT-PCR) was associated with *H. pylori-*positive non-atrophic gastritis, in both antral and oxyntic mucosa samples (*p*=0.011 and *p*=0.007, respectively; *t*-test; Figure 1A). For 10 patients enrolled as *H. pylori-positive*, paired biopsy samples were obtained after the bacterium's eradication; in 8 of these 10 cases, let-7c expression was significantly higher after the eradication treatment (*p*=0.009; *t*-test; Figure 1B).

The relationship between *H. pylori* infection and let-7c expression was further explored in the NCBI-GEO database and using the GEO2R microarray analysis tool. In 2 of the 3 available series, *H. pylori-*positive samples featured a significant let-7c down-regulation (GSE32174 [31], adjusted *p*=3.47E-02; and GSE19769 [20], adjusted *p*=4.3E-04; *t*-test) (Figure 1C–1D); in the third series, let-7c expression was still lower, though not significantly so, in *H. pylori-*positive than in normal mucosa (GSE54397 [32]; data not shown).

### Let-7c is down-regulated during *H. pylori*-related gastric carcinogenesis

Let-7c expression was tested (qRT-PCR) in 115 FFPE samples representative of normal mucosa and each of the phenotypic lesions occurring in the oncogenic cascade (normal antral mucosa, 20 cases; IM, 35 cases; LG-IEN, 15 cases; HG-IEN, 15 cases; intestinal-type GC, 30 cases).

**Figure 2:**
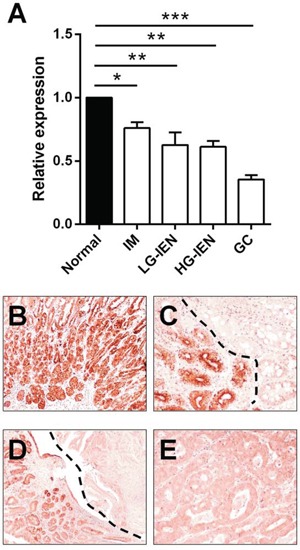
Let-7c is significantly down-regulated during *Helicobacter pylori*-related gastric carcinogenesis **A.** Let-7c expression, analyzed by qRT-PCR in 115 FFPE biopsy samples, gradually decreased along the *H. pylori-related* gastric carcinogenic cascade (*p*<0.001; relative expression= fold changes compared to normal antral mucosa samples set as 1.0). Compared with normal antral mucosa, let-7c was significantly down-regulated in intestinal metaplasia (IM; *p*=0.041), low-grade intraepithelial neoplasia (LG-IEN; *p*=0.005), high-grade IEN (HG-IEN; *p*=0.002), and intestinal-type gastric cancer (GC; *p*<0.001). (*=*p*<0.05; **=*p*<0.01 ***=*p*<0.001) **B–E.** ISH consistently revealed a strong let-7c expression in normal antral epithelia **B**, whereas samples of IM (**C**, top right), IEN (**D**, top right), and GC **E.** showed faint or no staining. (Original magnifications 10x and 20x)

Overall, let-7c expression decreased significantly with the increasing severity of the lesions considered (p<0.001; ANOVA; Figure 2A). Compared with normal *H. pylori*-negative antral mucosa, let-7c down-regulation was significant in IM (*p*=0.041; *t*-test), LG-IEN (*p*=0.005; *t*-test), HG-IEN (*p*=0.002; *t*-test), and GC (*p*<0.001; *t*-test).

These results were validated by testing let-7c expression (*in situ* hybridization) on FFPE samples obtained from 5 gastrectomy specimens of intestinal-type GC (Figure 2B–2E). In the multiple tissue samples obtained from each of the surgical specimens considered, strong let-7c hybridization signals were consistently found in native antral mucosecreting epithelia (Figure 2B), while a significant let-7c down-regulation was associated with intestinal metaplasia (Figure 2C), IEN (Figure 2D), and overt adenocarcinoma (Figure 2E).

**Figure 3:**
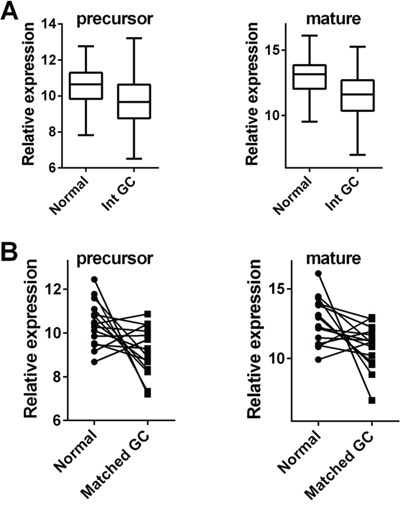
Let-7c is significantly down-regulated in intestinal-type gastric adenocarcinoma Let-7c expression was explored in intestinal-type GCs and matched normal gastric mucosa series profiled in The Cancer Genome Atlas (TCGA) [14]. **A.** The levels of both precursor and mature forms of let-7c were significantly lower in GC (*n*=177) than in normal gastric mucosa (*n*=41) (both *p*<0.001; relative expression= distribution of normalized data obtained from the TCGA database). **B.** In 15 TCGA patients with matched normal/GC samples, both the precursor and the mature let-7c form were down-regulated in the neoplastic samples (*p*=0.022 and *p*=0.029, respectively; relative expression= distribution of normalized data obtained from the TCGA database).

The above-mentioned results are basically consistent with the profile of let-7c (precursor and mature forms) in The Cancer Genome Atlas (TCGA). As emerged from the Atlas database, the levels of both precursor and mature forms of let-7c were significantly lower in cancer than in normal mucosa among 177 cases of intestinal-type GC, and 41 cases of normal gastric mucosa (both *p*<0.001; *t*-test; Figure 3A). Moreover, among the 15 TCGA cases of matched normal and GC samples, cancers featured let-7c down-regulation in its precursor (11/15) and/or mature form (13/15) (*p*=0.022 and *p*=0.029, respectively; paired *t*-test) (Figure 3B).

### In autoimmune gastritis, let-7c down-regulation is associated with intestinal type, but not pseudo-pyloric type metaplasia

As seen in the *H. pylori-*related gastritis model, corpus-restricted autoimmune gastritis may also result in the atrophic transformation of the native mucosa, often associated with metaplastic (pseudo-pyloric and intestinal) transformation of the resident specialized epithelia [25]. These lesions are currently considered at higher risk of cancer onset and frequently coexist with enterochromaffin-like (ECL) cell hyperplasia, both linear and micronodular (the precursor lesions of gastric carcinoids).

In this “autoimmune model”, let-7c status was tested to assess its potential involvement in the earliest phases of the morphogenesis involved in atrophic transformation unrelated to *H. pylori*.

**Figure 4:**
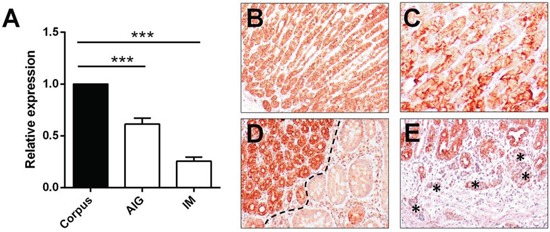
Let-7c is significantly down-regulated in autoimmune gastritis **A.** Let-7c expression was measured (qRT-PCR) in 40 FFPE corpus biopsy samples obtained from patients with autoimmune gastritis (20 cases of corpus-restricted non-atrophic gastritis [AIG] and 20 of intestinal metaplasia [IM]). Both groups showed a significant down-regulation of the miRNA by comparison with normal oxyntic mucosa (***= *p*<0.001; relative expression= fold changes compared to normal oxynthic mucosa samples set as 1.0). ISH for let-7c consistently showed strong staining in oxyntic mucosa **B**, and a weaker expression in parietal cells by comparison with other oxyntic cell types **C.** Strong let-7c expression was also apparent in pseudo-pyloric metaplastic epithelia (on left of panel), whereas a significant down-regulation was seen in IM **D.** ECL hyperplasia (asterisks) showed faint let-7c staining **E.** (Original magnifications 10x and 20x)

Let-7c expression was assessed by qRT-PCR in 40 FFPE corpus biopsy samples (20 cases of corpus-restricted non-atrophic gastritis, and 20 of intestinal metaplasia) obtained from patients with AIG and no history of *H. pylori* infection. Compared with normal oxyntic mucosa samples, a significant let-7c down-regulation was consistently associated with both non-atrophic AIG and AIG-related IM (both *p*<0.001; *t*-test) (Figure 4A).

Let-7c expression was further tested by *in situ* hybridization in a series of 15 FFPE samples representative of the histological spectrum of AIG (non-atrophic and atrophic variants). The biopsy samples included 5 cases of normal oxyntic mucosa, 5 of non-atrophic AIG, and 5 of oxyntic atrophic-metaplastic gastritis (including both intestinal and pseudo-pyloric metaplasia; Figure 4B–4E).

Let-7c hybridization consistently disclosed a normal miRNA expression in normal oxyntic glands (Figure 4B–4C), and metaplastic pseudo-pyloric epithelia, while a significant down-regulation was apparent in intestinalized cells (Figure 4D). Any ECL hyperplasia showed faint let-7c staining (Figure 4E).

## DISCUSSION

*Lethal-7* (*let-7*) is a founding member of the miRNA family of genes that was first identified in *Caenorhabditis elegans* [26–28]. Several homologs of *C. elegans let-7* have been recognized in humans, including *hsa-let-7c* [27]. Members of the human let-7 family share an identical seed sequence crucial to target recognition. They are distributed in 12 genomic loci known as breakpoint regions and fragile sites that are commonly affected in cancer [29, 30]. Several independent studies have indicated that *let-7* members act as tumor suppressors on various tumor types by targeting *KRAS* and *HMGA2* oncogenes [23, 31–35].

Like other malignancies, GC is consistently associated with *let-7* down-regulation [23]. In a large series of GCs, Motoyama *et al.* first demonstrated that let-7 family members negatively regulated *HMGA2* expression [23]. By profiling *H. pylori-*infected gastric mucosa using miRNA microarrays, Matsushima *et al.* demonstrated an important down-regulation (>2-fold) of 5 members of the let-7 family (let-7a, let-7b, let-7d, let-7e, let-7f) in *H. pylori-*positive mucosa [20]. In the 4 patients successfully treated to eradicate the bacterium, there was subsequently a significant increase in 14 miRNAs, including let-7a, let-7b, let-7d, and let-7e [20]. Similar results emerged from a comprehensive miRNA array analysis on gastric epithelial cells transfected with the *H. pylori* cagA gene [21]. In this *in vitro* model, Y. Hayashi et al. also found let-7 family down-regulation associated with an increased DNA-methyltransferase-DNMT3B activity, resulting in histone and DNA methylation of the *let-7* gene promoter (21).

As for the present study, we first explored *hsa-let-7c* expression in the whole spectrum of phenotypic changes occurring in the *H. pylori-related* carcinogenic cascade. In a large series of representative gastric mucosa biopsy samples, let-7c was found significantly down-regulated in *H. pylori-*infected mucosa samples (from the antrum and corpus), and its expression was restored after *H. pylori* eradication.

We demonstrated that let-7c expression drops significantly (qRT-PCR) since the earliest phenotypic changes (Intestinal Metaplasia) occurring in *H. pylori-*associated carcinogenesis and its loss of expression parallels the increasing severity of the histological changes. The significant miRNA down-regulation featured already in IM samples (ISH) is also consistent with a significant down-regulation of let-7c in serum, as recently reported in a large series of patients with atrophic gastritis (*n*=222), and GC (*n*=214) [22]. In line with our results, these data further support the candidacy of let-7c as a promising noninvasive biomarker in GC [22]. Next step will be the evaluation of both tissue and circulating let-7c expression levels in a large series of atrophic gastritis patients stratified according to different gastritis stages [11] to assess let-7c prognostic impact as a noninvasive biomarker in the progression from initial gastritis phases to cancer-prone multifocal gastritis.

We also tested let-7c expression in a model of autoimmune gastritis. In this setting, both non-atrophic and IM-atrophic biopsy samples showed a significant miRNA down-regulation compared with normal oxyntic mucosa (both *p*<0.001). The significant down-regulation observed on ISH in IM (but not in pseudo-pyloric metaplasia) suggests a role for let-7c in driving the earliest changes occurring in the gastric carcinogenic cascade, while it does not support any elective oncogenic influence of pseudo-pylorized metaplastic cells [25, 36–38].

A recent study [39] pinpointed the significant role of HMGA2, a let-7c target, in the progression and prognosis of GC through the activation of the epithelial-mesenchymal transition process in GC cells. These data and the present results (i.e., let-7c expression decreased significantly with the increasing severity of the lesions considered) support an important role of let-7c in both the initiation and progression of the gastric neoplastic transformation.

In conclusion, our findings in a large series of histologically well-profiled phenotypic lesions demonstrate that let-7c down-regulation plays a pathogenic part right from the earliest phenotypic changes taking place in the *H. pylori-related* carcinogenic process, and further supports the tumor suppressor activity of this miRNA family. The reversibility of let-7c down-regulation, as seen after *H. pylori* eradication, also testifies to a “functional plasticity” of this potential candidate for use as an adjunctive test on gastric mucosa status. We also demonstrated for the first time that let-7c is significantly down-regulated even in early cancer precursor lesions occurring in autoimmune gastric oncogenesis. Overall, these results provide a solid rationale for further exploring the diagnostic reliability of this miRNA family as a novel biomarker for use in GC secondary prevention strategies.

## MATERIALS AND METHODS

### Ethics statement

Investigation has been conducted in accordance with the ethical standards and according to the Declaration of Helsinki and according to national and international guidelines and has been approved by the authors' institutional review board (#0012272/15).

### Tissue samples

The cases considered in this study were retrospectively collected from the files of the Surgical Pathology and Cytopathology Unit of the Department of Medicine (DIMED - University of Padua). The Institute's ethical regulations concerning research conducted on human tissues were followed.

A total of 195 endoscopic formalin-fixed paraffin-embedded (FFPE) biopsy samples were considered in the let-7c qRT-PCR analysis. The tissue samples were selected from 140 different biopsy sets obtained from 130 Caucasian patients (M/F: 1.5/1; mean age 63.2±8.4 years) who underwent gastro-esophageal endoscopy at the Gastroenterology Unit of the Department of Surgical Oncology and Gastroenterology (DiSCOG, University of Padua).

Biopsy samples were representative of each of the phenotypic lesions in the *H. pylori-*related gastric carcinogenic cascade [11, 40–43]. They included: i) 40 normal gastric mucosa samples (20 antral, 20 oxyntic) from 20 patients undergoing endoscopy for functional dyspepsia; ii) 40 *H. pylori-*positive non-atrophic gastritis samples (20 antral, 20 oxyntic) from 20 patients (whose *H. pylori* status was assessed histologically by means of Giemsa staining); iii) 20 antral mucosa samples obtained from 10 patients before and after *H. pylori* eradication therapy (median follow-up: 10 weeks; *H. pylori* eradication was always confirmed with the 13C-urea breath and/or stool antigen tests); iv) 35 antral mucosa samples with extensive intestinal metaplasia (IM; histochemically sub-typed as Type II-III IM by means of high iron diamine staining) obtained from patients with OLGA stage III and IV gastritis; v) 15 low-grade intra-epithelial neoplasia samples (LG-IEN; formerly called ‘low-grade dysplasia’); vi) 15 high-grade IEN samples (HG-IEN; formerly ‘high-grade dysplasia’); vii) 30 well- or moderately-differentiated intestinal-type GC samples (all G1/G2, and all pT1 or pT2).

A further 40 FFPE oxyntic mucosa samples were obtained from 40 Caucasian patients (M/F: 0.8/1; mean age 55.2±9.0 years) with a clinical history of autoimmune gastritis (AIG). In all cases, their autoimmune etiology was assessed histologically and confirmed by serologically testing anti-parietal cells and/or anti-intrinsic factor autoantibodies (Autozyme IFAb; Cambridge Life Sciences, Ely, UK) [13]. These biopsies included: i) 20 samples of non-atrophic corpus-restricted gastritis; ii) 20 oxyntic mucosa samples with extensive IM obtained from OLGA stage II and III autoimmune gastritis.

*In situ* hybridization (ISH) was performed on FFPE tissue samples obtained from 5 gastrectomy specimens for adenocarcinoma (all distal cancers associated with ongoing or previous *H. pylori* infection); and 10 biopsy samples of oxyntic mucosa obtained from AIG patients were also included. Where indicated, tissue samples were micro-dissected from the formalin-fixed paraffin-embedded tissues so that the tested tissue samples always included at least 70% of the target lesion (as further validated on serial H&E histology sections). All histological assessments were performed jointly by three pathologists (MF, DS, IC).

### Reverse transcription and quantitative real-time PCR

Total RNA was extracted using the RecoverAll kit (Ambion, Austin, TX, USA). The NCodeTM miRNA qRT-PCR method (Invitrogen, Carlsbad, California, USA) was applied to detect and quantify mature hsa-let-7c according to the manufacturer's instructions, using an ABI Prism 7900HT Sequence Detection System (Applied Biosystems, Foster City, CA) [24, 44]. Normalization was done with the small nuclear RNA U6B. All reactions were run in duplicate, including no-template controls. The fold difference for each sample was obtained using the ΔΔCT method.

### Let-7c *in situ* hybridization (ISH)

Locked nucleic acid (LNA) probes with complementarity to let-7c were labeled with 5′-biotin and synthesized using Exiqon (Vedbaek, Denmark). Tissue sections were digested with ISH protease 1 (Ventana Medical Systems, Milan, Italy) and ISH was performed as previously described [45], with minor modifications. Positive (U6; Exiqon) and negative scrambled LNA probes were used as controls. Only cytoplasmic let-7c intensity was retained for scoring purposes.

### Array database meta-analysis

The NCBI-GEO repository of published array data and the GEO2R microarray analysis tool were used (15 June 2015) to assess let-7c expression in *H. pylori-related* gastritis (using the keywords: microRNA, gastritis, *Helicobacter pylori*, *Homo sapiens*, gastric). The GEO2R algorithms were used for the statistical analysis on the differences in let-7c expression between the groups (normal gastric mucosa *vs* Hp-gastritis).

To test let-7c dysregulation in gastric cancer, let-7c expression was explored among intestinal-type gastric adenocarcinomas and matched normal gastric mucosa series profiled in The Cancer Genome Atlas (TCGA) initiative [14]. Normalized let-7c expression data were used in the analysis.

### Statistical analysis

Differences between groups were tested by applying the (paired) *t*-test, and ordinary one-way ANOVA, as appropriate. P values <0.05 were considered significant. The statistical analysis was performed using STATA software (Stata Corporation, College Station, TX).
